# Investigation of kinetic, isotherm and adsorption efficacy of thorium by orange peel immobilized on calcium alginate

**DOI:** 10.1038/s41598-023-35629-z

**Published:** 2023-05-24

**Authors:** Ali A. Gh. Khamseh, Sohrab Ali Ghorbanian, Younes Amini, Mohammad Mahdi Shadman

**Affiliations:** 1 Nuclear Fuel Cycle Research School, Nuclear Science and Technology Research Institute, Tehran, Iran; 2 Faculty of Chemical Engineering, School of Engineering, University of Tehran, Tehran, Iran

**Keywords:** Chemistry, Energy science and technology, Engineering

## Abstract

In this research work the thorium uptake on immobilized protonated orange peel was studied in a batch system. The effects of effective parameters such as biosorbent dosage, initial metal ion concentration, and contact time on the biosorption of thorium were analyzed. The biosorption capacity of the immobilized orange peel for thorium at optimal conditions of initial pH 3.8, biosorbent dosage 8 g/L, and initial thorium concentration 170 mg/L was found to be 18.65 mg/g. According to the results of contact time, the biosorption process reached equilibrium after around 10 h of contact. Investigation of the kinetics showed that the biosorption of thorium onto immobilized orange peel follows the pseudo-second-order model. The Langmuir and Freundlich isotherms were used to model the experimental equilibrium data. The results showed better agreement by the Langmuir isotherm. The maximum absorption capacity of immobilized protonated orange peel for thorium adsorption was predicted by the Langmuir isotherm at 29.58 mg/g.

## Introduction

Heavy metals are one of the most important environmental concerns due to their toxic effects on plants, animals and humans^1–4^. Uranium mining processes and uranium–thorium ore mining units, burning brown coal in coal-fired power plants, and using chemical fertilizers generate large volumes of thorium-containing wastewater every year. Removing thorium from aqueous solutions is necessary due to environmental and human health issues^5–8^. Since thorium is toxic like most other heavy metals, releasing solutions containing thorium into the environment is considered a serious risk to human life and other living organisms. For this reason, the need to remove and recycle this element with a cost-effective method is felt.

There are various common methods for treating wastewater containing heavy metal ions, including thorium, which include chemical precipitation^9,10^, oxidation or reduction^11,12^, membrane technologies^13,14^, filtration, flotation^15,16^, electrochemical treatment^17–19^, reverse osmose^20^, and solvent extraction^21–26^, ion exchange^27–29^. Most of these methods involve high capital costs or are only suitable for separating high concentrations of heavy metals. Adsorption is very cheap and has very good flexibility in adsorbing heavy metals^30–35^. Some of the adsorbents used in the adsorption of heavy metals are activated carbon, ion exchange resins and biological adsorbents^36–42^. Biological adsorption as a cheap and effective method in the treatment of industrial wastewater has attracted the attention of specialists. The process of biological adsorption is the removal of heavy metals and other pollutants from the environment through their adsorption on non-living microorganisms and other organic materials (such as algae, fungi, rice straw, fruit peel, etc.)^43,44^. The advantages of the biological adsorption process in comparison with other common methods of removing heavy metals are economic efficiency, the ability to regenerate and reuse the adsorbent in successive cycles, the possibility of recycling metals, the high speed of the process, and the absence of sludge production^45–48^.

Some researchers used a variety of adsorbents for thorium removal. Anirudhan et al.^5^ used poly (methacrylic acid)-grafted chitosan/bentonite composite, Soltani et al.^49^ used multi-walled carbon nanotube, Xu et al.^50^ used magnetic chitosan resins, Khalili et al.^51^ used humic acid, Khamseh et al.^52,53^ used orange peel, Xiu et al.^54^ used graphene oxide nanoribbons/manganese dioxide composite, Hu et al.^55^ used Cu 3(BTC)2, Huang et al.^56^ used mesoporous graphite carbon nitride, Akl et al.^57^ used amidoximated copolymeric hydrogel, and Liu et al.^58^ used a three-dimensional covalent organic framework.

Despite the extensive study for thorium adsorption, thorium adsorption using orange peel immobilized on calcium alginate has not been reported.

Orange peel compounds include pectin, cellulose, hemicellulose, and limonene, which have carboxyl, hydroxyl, etc. functional groups and have a high affinity with metals^52,53^. The use of orange peel in the column of fixed beds causes problems in the process due to swelling, clumping, channeling, and Finally, column blockage^53^. The immobilization of biological adsorbents using natural and synthetic polymer substrates is used to solve this problem. Immobilized biosorbents offer advantages such as better reusability, higher biomass loading, less clumping and no column blockage in fixed bed systems and relatively high local density. By immobilizing the orange peels on alginate and forming stabilized adsorbent beads, the problem of swelling and column breakage is solved.

In this research work, for the first time, thorium adsorption ability using orange peel immobilized on calcium alginate as a cheap and affordable adsorbent in terms of economy and process has been investigated in a batch. In addition, the effect of effective parameters on the adsorption process, such as initial metal concentration, biosorbent dosage, and contact time on thorium adsorption, as well as kinetic studies of thorium adsorption and adsorption equilibrium isotherms have also been studied.

## Materials and methods

### Materials

Thomson cultivar orange peel from northern Iran was used to prepare the adsorbent. Sodium alginate ((C_6_H_7_O_6_Na)_n_) was purchased from Sigma Aldrich, thorium nitrate (Th(NO_3_)_4_⋅5H_2_O) and other chemical solutions were purchased from Merck in analytical grades.

### Adsorbent preparation

**Figure 1:**
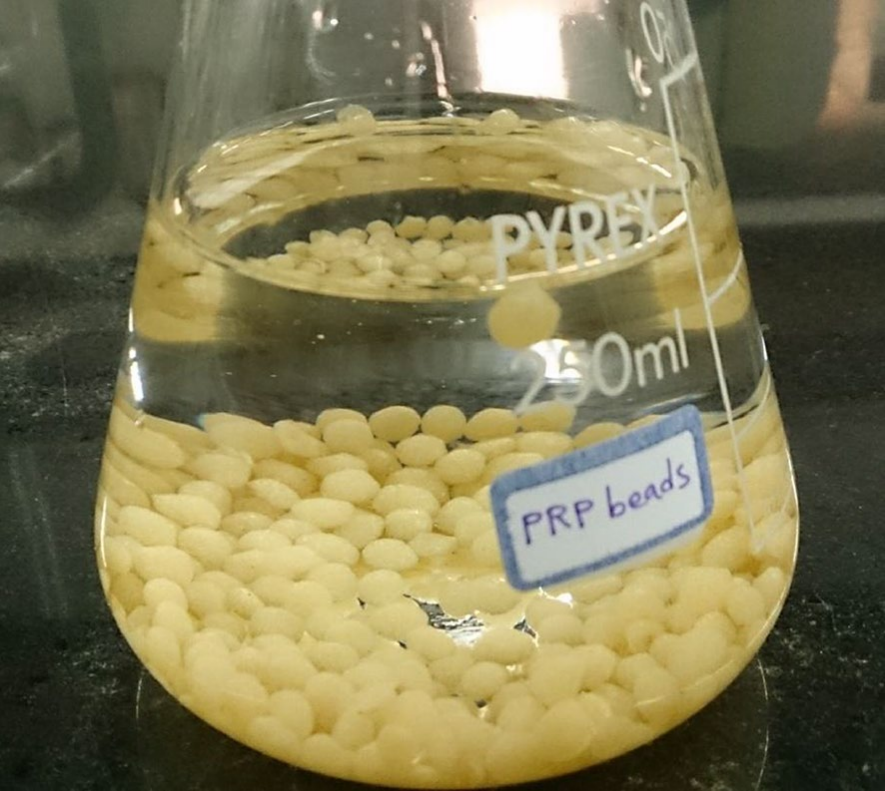
Beads of immobilized POPs.

To prepare the adsorbent, raw orange peels were cut into 1–3 mm sizes and washed. The amount of 10 g of these raw peels was poured into 500 mL of 0.1 normal nitric acid and the suspension was stirred for 2 h at a constant speed of 150 rpm at 25 °C until the active adsorbent sites were saturated with H^+^ ions. From this point on, these peels are called protonated orange peels (POP). Protonation with nitric acid has been done to remove naturally present ions from the orange peel and thus obtain a better chemically modified orange peel where all weakly acidic sites are occupied by protons (H^+^)^52,59,60^. The suspension was filtered and then the POPs were washed with distilled water and dried in an oven at 45 °C for 24 h. To perform the immobilization process, first, the protonated and dried raw peels were ground and sieved with 180 μm mesh. Then a suspension of 0.5 g of it was prepared along with 20 mL of 1.5% sodium alginate solution at a ratio of 2:1. This solution is added dropwise in 2.5% calcium chloride solution using a syringe to form orange peel solid beads in calcium alginate^60^. The formed beads are shown in Fig. 1. The immobilized biosorbent in this solution (≈ 3 mm) was placed in a refrigerator at 4 °C for 4 h. These immobilized orange peel beads are kept in distilled water solution for use in subsequent experiments.

### Adsorbent characterization

The morphology of the beads before and after adsorption was investigated by Field Emission Scanning Electron Microscopy (FESEM) (Hitachi S4160). To prepare specimens for the FESEM, samples were dehydrated and then dried and sputter coated with gold.

### Adsorption experiments

Thorium biosorption experiments were carried out by immobilizing orange peels in 100 mL Erlenmeyer flasks with 20 mL of thorium nitrate solution with a certain concentration and optimal pH of 3.8^52^ using an adsorbent with a certain concentration. For temperature uniformity (25 °C) and circular motion, the solutions were placed in a shaker incubator (Gallenkamp) at the stirring speed of 150 rpm, and at certain times, i.e. 2, 5, 10, 15, 20, 60, 120, 240, 360, 480, and 1440 min after the start of the adsorption process, samples were taken from the solution and the samples were sent for analysis. ICP model Optima 2000DV was used to determine thorium concentration in the samples.

In order to investigate the effect of operating parameters on the adsorption of thorium by the adsorbent, first the equilibrium contact time was determined, then the experiments necessary to determine the effect of the parameters of the amount of adsorbent using adsorbent dosage of 2, 4, 6, 8, and 10 g/L and the initial concentration of thorium was 100, 150, 200, 250, 300, and 350 mg/L. To reduce the error, all experiments were repeated three times and the average of obtained data was utilized.

For data processing and modeling, the thorium removal efficiency was determined using Eq. (1)^55^.

1 $${\mathrm{Removal efficiency} }\left({\%}\right)=\frac{{C}_{i}-{C}_{f}}{{C}_{i}}\times 100$$

where C_i_ is the initial concentration (mg/L) and C_f_ is the final concentration (mg/L) of thorium ions. The term q is defined as the amount of ion adsorbed on a certain amount of adsorbent (mg/g). Adsorption capacity at time t, q_t_ (mg/g) is expressed as Eq. (2)^55^.

2 $${q}_{t}=\frac{\left({C}_{i}-{C}_{t}\right)V}{m}$$

where C_i_ and C_t_ (mg/L) are the concentrations of the liquid phase of the dissolved substance at the initial time and desired time t, V is the volume of the solution and m is the mass of the adsorbent (g). The amount of adsorption at equilibrium, q_e_ (mg/g), is obtained by Eq. (3)^55^.

3 $${q}_{e}=\frac{\left({C}_{i}-{C}_{e}\right)V}{m}$$

C_e_ (mg/L) is the ion concentration at the time of equilibrium.

## Results and discussion

### Biosorbent characterization

**Figure 2:**
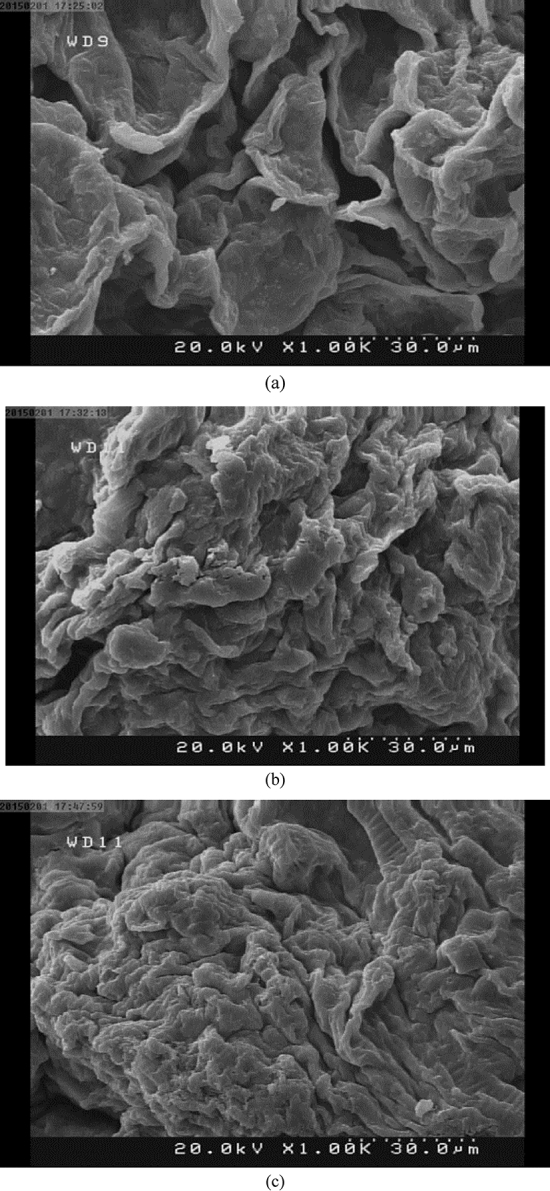
FESEM images at a magnification of $\times$1000 (**a**) POP before immobilization, (**b**) immobilized beads before adsorption, and (**c**) immobilized beads after adsorption.

The FESEM graphs of POP before and after immobilization, as well as before and after the adsorption of thorium ions, are shown in Fig. 2. According to this figure, whether before immobilization (a) or after immobilization (before (b) and after adsorption (c)), the surface structure of POP grains is non-uniform and porous. This structure shows that there is a good substrate for the adsorption of ions on the adsorbent surface. By comparing figures (a) and (b), the immobilization effect is clearly visible. On the other hand, as it is clear from the comparison of the figures before (b) and after adsorption (c), the adsorbent surface after adsorption has a more uniform structure than before adsorption, which indicates thorium ions sitting on the adsorbent sites (pores).

### The effect of adsorbent dosage

**Figure 3:**
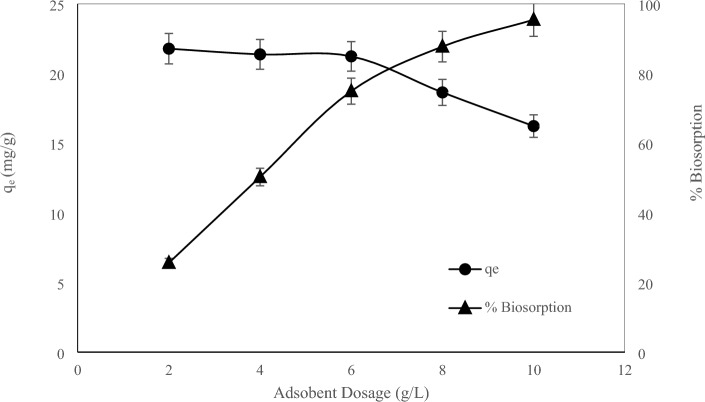
Effect of adsorbent dosage on adsorption rate and biosorption percentage (C_i_ 170 mg/L, pH 3.8, T 25 °C, and t 24 h).

The effect of the amount of adsorbent in the range of 2 to 10 g/L on the adsorption rate and removal percentage of thorium ions was investigated and the results are shown in Fig. 3. According to the figure, the adsorption capacity has decreased with the increase in the amount of adsorbent from 21.79 to 16.23 mg/g. According to relations (2) and (3) the amount of adsorption is defined based on the amount of adsorbent unit weight, as the amount of biosorbent increases, the amount of adsorption per unit mass of adsorbent decreases. Another reason for this is remaining unsaturated sites during the adsorption process with increasing adsorbent dosage. On the other hand, according to the figure and based on Eq. (1), with the increase in the amount of biosorbent, the percentage of biosorption (removal efficiency) increases from 25.64 to 95.47%. Since the concentration of the adsorbent indicates the number of available sites of the adsorbent for the adsorption of heavy metals, and due to the increase in the amount of the adsorbent, more contact surface of the adsorbent is available, therefore more sites are available for the adsorption of metal ions. With more increase in the amount of adsorbent, the upward trend of the adsorption percentage slows down, because the concentration of metal ions adsorbed on the surface of the adsorbent and the concentration of metal ions in the solution reach a balance, and therefore, the more the amount of adsorbent increases, the adsorption percentage does not increase. Therefore, the amount of adsorbent used should be optimized. According to Fig. 3, the optimal amount of biosorbent was determined to be 8 g/L, and this dosage was used through experiments.

### The effect of contact time

**Figure 4:**
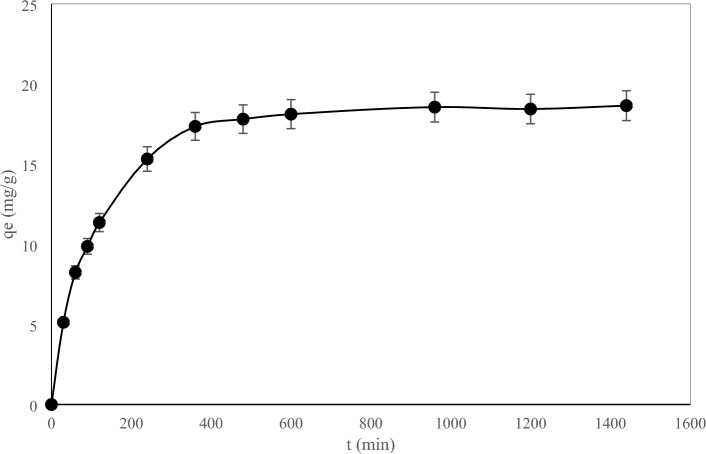
The effect of contact time on the adsorption rate in the biological adsorption of thorium (C_i_ 170 mg/L, adsorbent dosage 8 g/L, pH 3.8, and T 25 °C).

**Figure 5:**
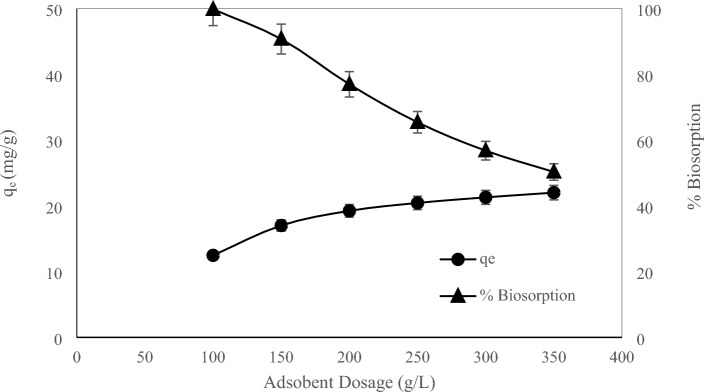
The effect of the initial concentration of thorium on the amount and percentage of biosorption (adsorbent dosage 8 g/L, T 25 °C, and pH 3.8).

The effect of contact time on the adsorption of thorium concentration in the solution was done at time intervals of 30, 60, 90, 120, 240, 360, 480, 600, 960, 1200, and 1440 min after contact with the adsorbent. The time required to reach equilibrium was also determined in this phase of the experiments. The time required to establish equilibrium between the metal cations adsorbed by the adsorbent sites and the remaining ions in the solution is called the equilibrium time. According to Fig. 4, the amount of adsorption increases with a steep slope at first, so that more than 50% of the adsorption has taken place in the first 90 min of contact, then it proceeds with a gentle slope until it reaches equilibrium. In the first minutes, due to the fact that most of the adsorption sites are empty, the chemical potential difference between the adsorbent and the solution is high, and therefore, the driving force of the mass transfer is high and the adsorption speed is high. But with the passage of time and the gradual filling of the adsorption sites, the chemical potential difference between the adsorbent and the solution decreases and causes the adsorption rate to decrease and finally reach a constant value. Therefore, according to these results and matching Fig. 5, the equilibrium time can be considered to be 10 h.

### The effect of initial concentration

A series of adsorption experiments with initial concentrations of 100, 150, 200, 250, 300, and 350 mg/L of thorium solution at optimal pH of 3.8 and using 8 g/L of immobilized adsorbent at 25 °C for 24 h, to investigate the effect of the initial concentration on the adsorption capacity and the removal percentage of thorium metal ions, (Fig. 5). According to the figure, as the initial concentration of the solution increases, the amount of adsorption increases, but the percentage of adsorption decreases. By increasing the initial concentration of a solution from 100 to 350 mg/L, the amount of thorium adsorbed increases from 12.48 to 22.02 mg/g, and the adsorption percentage decreases from 99.84 to 50.33%. The concentration difference between thorium in solution and adsorbent is the driving force of adsorption. Therefore, by creating a difference in concentration, a higher amount of adsorption can be achieved. Hence, the higher initial concentration of thorium in the solution will improve the driving force and thus the adsorption rate. As the initial concentration of the solution continues to increase, due to the fact that at high concentrations, the adsorption sites on the biosorbent are filled, and as a result, according to the form of adsorption, it reaches an almost constant amount. In fact, the increase in adsorption capacity is due to the availability of more biosorbent sites for the adsorption of thorium ions, which in high concentrations of the solution, the approximate stabilization of the adsorption capacity is due to the saturation of the active sites of the adsorbent^61,62^. Also, according to Fig. 5 and the definition of adsorption percentage based on Eq. (1), the adsorption percentage decreases as the initial concentration of the solution increases.

### Adsorption kinetics

The kinetics of the adsorption process provides essential information about the reaction path and the rate of the adsorbate. Pseudo-first-order and pseudo-second-order kinetic models are among the most common kinetic models that are used in examining the experimental data of heavy metal adsorption kinetics on adsorbents. The linearized pseudo-first-order kinetic model can be described according to Eq. (4)^55^.

4 $$\mathrm{ln}\left({q}_{e}-{q}_{t}\right)=\mathrm{ln}{q}_{e}-{k}_{1}t$$

where q_e_ and q_t_ are the adsorption capacity (mg/g) at equilibrium time and time t, respectively, while k_1_ (1/min) shows the pseudo-first-order rate constant.

**Figure 6:**
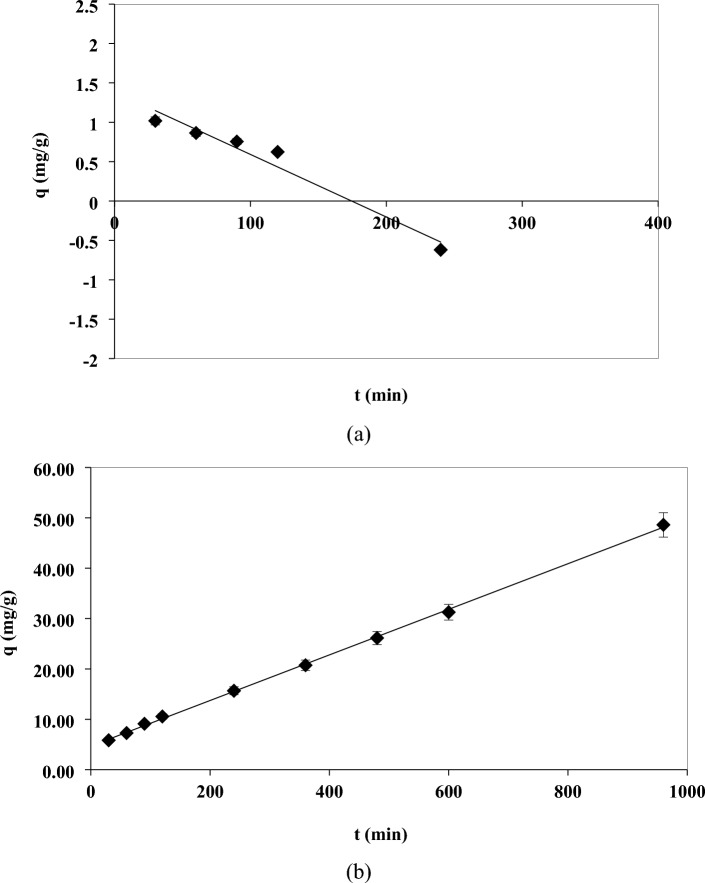
Linearized kinetic equations of pseudo-first order (**a**) and pseudo-second-order (**b**) adsorption of thorium ions by immobilized POP (C_i_ 170 mg/L, adsorbent dosage 8 g/L, T 25 °C, and pH 3.8).

The adsorption kinetics can also be described by a pseudo-second-order model. The linearized equation of this model is expressed as follows^55^:

5 $$\frac{t}{{q}_{t}}=\frac{1}{{k}_{2}{q}_{e}^{2}}+\frac{1}{{q}_{e}}$$

where k_2_ (g/mg.min) is the pseudo-second-order rate constant and q_t_ and q_e_ are the adsorption capacity (mg/g) at time t and equilibrium time, respectively. Figure 6a shows the linearized kinetic equation of Pseudo-first order and experimental data of kinetics of thorium adsorption. The parameters of the pseudo-first-order kinetic model, k_1_ and q_e_ were 0.0034 (1/min) and 12.34 (mg/g), respectively, obtained from linear fitting with a correlation coefficient of 0.9584, which shows that the model pseudo-first order is not a suitable model to describe the adsorption of thorium by immobilized POP. Figure 6b shows the linearized pseudo-second-order kinetic equation and the experimental data of thorium adsorption. The parameters of the pseudo-second-order kinetic model, k_2_ and q_e_ were 0.000438 (g/mg.min) and 22.075 (mg/g) respectively, obtained from linear fitting with a correlation coefficient of 0.9993, which shows that the pseudo-second-order kinetic model is a very suitable model for describing thorium adsorption by immobilized POP. Also, according to Fig. 6b, the predicted q_e_ value (22.075) by the pseudo-second-order kinetic model is much closer to the experimental qe value, i.e., 20.22, compared to the predicted q_e_ value (12.34) by the pseudo-first-order kinetic model (Fig. 6a). According to the very good fitting results with the pseudo-second-order kinetic model, it can be concluded that the rate-limiting step of the thorium removal process may be due to chemisorption processes^63^.

### Adsorption isotherms

In order to design an adsorption system for the separation of metal ions, it is necessary to find a suitable relationship (isotherm) to describe the results of equilibrium experiments in biosorption. In this research, Langmuir, Freundlich, and Dubinin-Radushkevich equilibrium isotherms have been studied to fit the equilibrium data.

The Langmuir isotherm is used for dynamic equilibrium surface adsorption on completely homogeneous surfaces and assumes a single-layer coating for the adsorbent surface and does not consider any interaction between the adsorbed molecules, and as a result, the adsorbent surface is considered homogeneous in terms of adsorption energy. The linearized form of this model is expressed by Eq. (6)^55,64^.

6 $$\frac{{C}_{e}}{{q}_{e}}=\frac{1}{{bq}_{m}}+\frac{{C}_{e}}{{q}_{m}}$$

where q_e_ is the amount of adsorbed metal per specific amount of adsorbent in mg/g, C_e_ is the equilibrium concentration of the solution in mg/L, b is the Langmuir constant in L/mg and q_m_ is the maximum amount of adsorbent capacity in mg/g is. To check the desirability of the adsorption process, the dimensionless parameter R_L_ is defined according to the following equation, which is called the separation constant:

7 $${R}_{L}=\frac{1}{{1+bC}_{i}}$$

where C_i_ is the maximum initial concentration of the adsorbed component in mg/L. The value of R_L_ determines the type of isotherm. If R_L_ is greater than one, adsorption is unfavorable; While R_L_ is equal to a linear isotherm, 0 < R_L_ < 1 indicates favorable adsorption and R_L_ equal to zero indicates irreversible adsorption^65^.

Freundlich isotherm describes the adsorption on heterogeneous surfaces. In this model, it is assumed that the surface of the adsorbent has different adsorption centers with different inclinations and at first, the stronger adsorption centers are filled and the others are filled in the order of their strength. This isotherm considers surface adsorption in the form of multilayers. The linearized form of this model is expressed by Eq. (8)^55,66^.

8 $$\mathrm{ln}{q}_{e}=\mathrm{ln}{k}_{f+}\frac{1}{n}\mathrm{ln}{C}_{e}$$

where k_f_ (L/g) is the Freundlich constant which is related to the adsorption capacity, and n is the dimensionless Freundlich constant which is related to the bond strength. The higher the n i.e. 1 < n < 10, indicates a favorable sorption process and the stronger the attraction between the adsorbent and the adsorbate.

Dubinin-Radushkevich isotherm is valid in the range of low concentrations and can be used to describe adsorption on both homogeneous and non-homogeneous surfaces. The linear form of this model is as follows^67,68^:

9 $$\mathrm{ln}{q}_{e}=\mathrm{ln}{q}_{m}-\beta {\varepsilon }^{2}$$

In this equation, β is the isotherm constant in terms of mol^2^/kJ^2^, which is related to the adsorption energy, and ε is the Polanyi adsorption potential (J/mol), which is defined according to the following relation:

10 $$\upvarepsilon =\mathrm{RTln}(1+\frac{1}{{C}_{e}})$$

In this equation, R is the global gas constant (8.314 J/mol K) and T is the temperature in Kelvin.

To determine the adsorption mechanism using the D-R isotherm, parameter E can be used.

11 $$\mathrm{E}=\frac{1}{\sqrt{2\beta }}$$

This parameter indicates the change of free energy in terms of kJ/mol, which is needed to transfer one mole of adsorbate from an infinite distance in the solution to the surface of the adsorbent. When E is smaller than 8 kJ/mol, physical adsorption will be the dominant mechanism, and if E is between 8 and 16 kJ/mol, the ion exchange mechanism will be dominant^67^.

**Figure 7:**
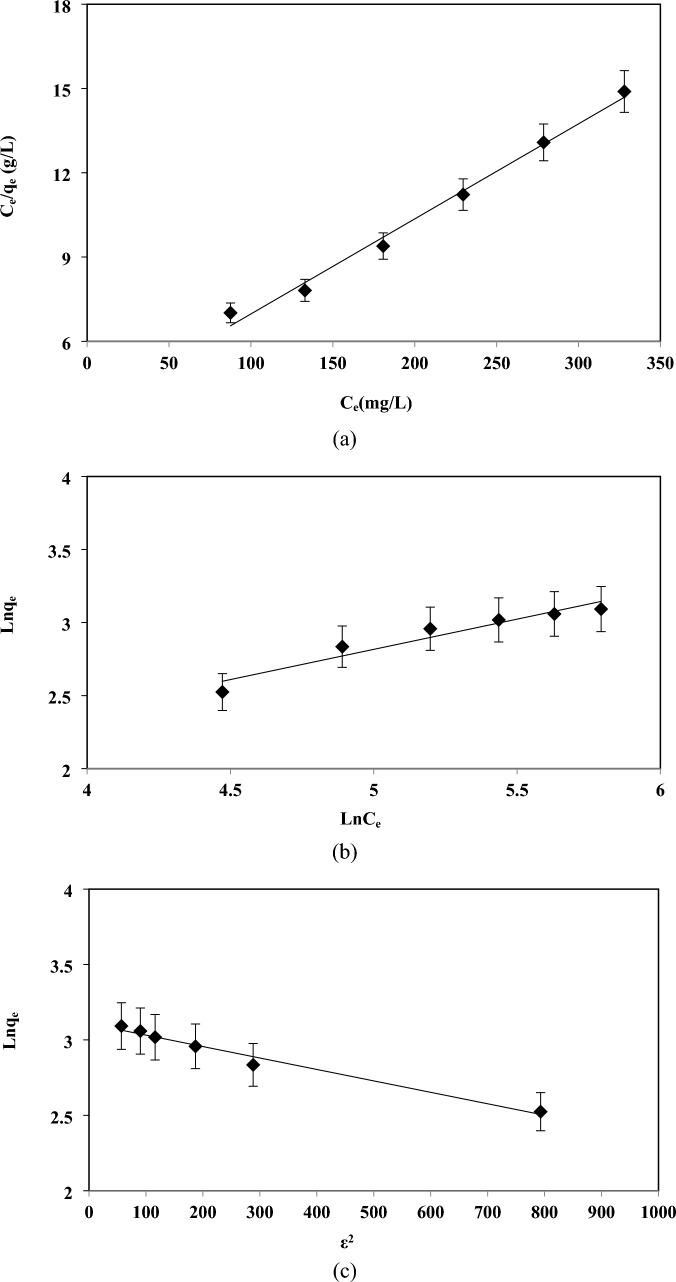
Langmuir (**a**), Freundlich (**b**), and Dubinin–Radushkevich (**c**) adsorption isotherms of thorium ions by immobilized POP (C_i_ 170 mg/L, adsorbent dosage 8 g/L, T 25 °C, and pH 3.8).

The diagrams of these three models' results are presented in Fig. 7a–c, and Table 1. According to the figure and the data in the table, it is clear that the Langmuir model has better compatibility with the experimental data. From this point, it can be concluded that the adsorption mechanism can be considered a single-layer and the adsorbent surface is homogeneous in terms of adsorption energy. The Langmuir model predicts the maximum adsorption rate of 29.58 mg/g adsorbent. According to what was stated before, the parameters of Langmuir and Freundlich isotherms can be used to predict the desirability of adsorption on the adsorbent. For the Langmuir isotherm, the value of the dimensionless parameter R_L_ for the range of initial concentration used (100 to 350 mg/l) has been calculated in the range of 0.5150 to 0.2328 respectively, which can be claimed that thorium adsorption on the immobilized orange peel adsorbent is favorable^69,70^. Also, the value of parameter n in the Freundlich isotherm is between one and ten, which indicates the optimal adsorption of thorium on the adsorbent^71,72^. The E value of the Dubinin–Radushkovic model, 0.025 kJ/mol (less than 8 kJ/mol), is another obvious indication that the adsorption mechanism is physical adsorption^67,68^.

**Table 1:** Adsorption isotherm parameters for thorium biosorption on immobilized POP.

Langmuir constants					Freundlich constants		Dubinin–Radushkevich constants			
q_m_ (mg/g)	b (L/mg)	R^2^		k_f_ (L/g)	n	R^2^	q_m_ (mg/g)	β (mol^2^/kJ)	E (kJ/mol)	R^2^
29.5858	0.009416	0.9904		2.1134	2.4178	0.926	22.37	0.0008	0.025	0.9798

### The effect of immobilization

According to the previous research done by the authors to adsorb thorium using orange peel as an adsorbent in a fixed bed column^53^, a series of problems such as adsorbent clumping and blocking of the column occurred during the process^53^. To avoid this problem, in this research, the technique of immobilization of POP on alginate was used.

Furthermore, according to other authors' research^52^, by using orange peel as an adsorbent in a batch system, the maximum adsorption capacity was determined to be 236.97 mg/g of adsorbent, while this value was 29.58 mg/g in this study. It was found that it shows a decrease in the amount of adsorption in the case of using the immobilization technique. The reason for this finding is that in using immobilized adsorbent, a smaller amount of the POP material is used compared to using POP without immobilization, so the adsorption capacity will be less. Also, the presence and confinement of tiny air holes inside the beads, which is somewhat unavoidable in the immobilization technique, reduces the effective volume of mass transfer, which can be one of the causes of the decrease in adsorption. In the case of using orange peel, the time to reach adsorption equilibrium was about 4 h, while in the present study, the equilibrium time was determined to be about 10 h. This finding shows a decrease in the speed of adsorption in the case of using the immobilization technique. This finding has also been observed by other researchers^73–75^. This phenomenon can be due to the larger size of the immobilized beads compared to POP powder. Another reason may be due to the necessity that the adsorbate must pass through the alginate layers to reach the functional groups of the adsorbent and be adsorbed on the immobilized adsorbent beads, and this itself causes a decrease in the speed of adsorption compared to the case of using unimmobilized POP powders.

It can be concluded that although in a batch system, the use of immobilized POP results in a lower absorption capacity and a slower rate of absorption than in the case of unimmobilized POP powder, the necessity of using the immobilization technique due to a series of limitations, including the difficulty of separating the adsorbent from the metal solution and the possibility of reusing it in an unimmobilized state, and especially the limitation of clumping and blocking of the column in the case of using unimmobilized POP in a fixed bed continuous column, the best reason for the superiority of using immobilized POP is established in the industry.

## Conclusion

In the current research, the adsorption of thorium (IV) on immobilized protonated orange peel was investigated in a batch system. The effects of three independent process variables including biosorbent dosage, Initial thorium concentration, and contact time on the biosorption of thorium were assessed. Based upon the obtained results, the optimal operating conditions of thorium adsorption were at an initial thorium concentration of 170 mg/L, and biosorbent dosage of 8 g/L in which under these optimal conditions, the thorium adsorption capacity was 18.65 mg/g. Moreover, the kinetics of the adsorption of thorium adsorption onto immobilized protonated orange peel followed the pseudo-second-order model. The biosorption process reached equilibrium after around 10 h of contact. The Langmuir equation provided good agreement for the experimental data. From the Lanmuir model results the maximum adsorption capacity of the immobilized POP was 29.58 mg/g in comparison to 236.97 mg/g in the case of unimmobilized POP. Even though the use of immobilized POP for thorium absorption had a lower absorption capacity than unimmobilized POP powder, due to overcoming a series of limitations such as clumping and blocking of the column in the case of using immobilized POP adsorbent in a fixed bed, immobilization of the POP has their own advantages in industry.

## Data Availability

The datasets used and/or analyzed during the current study are available from the corresponding author on reasonable request.
